# Beyond traditional library spaces: the practicalities of closing hospital libraries and opening a virtual library

**DOI:** 10.29173/jchla29434

**Published:** 2021-04-02

**Authors:** Carol A. Cooke

**Affiliations:** Health Sciences Liaison Librarian, Neil John Maclean Health Sciences Library, University of Manitoba Winnipeg, MB

## Abstract

**Introduction:**

The closure of hospital libraries is a noteworthy trend taking place across North America. A Canadian university and its affiliated health authority chose to close eight hospital libraries and merge them into one virtual library service based on changing use of library services, technology and budgetary concerns. This case study describes the processes and considerations both for closing library spaces and transitioning to a new virtual library service.

**Description:**

Project management processes efficiently guided the project to completion. These processes included stakeholder consultation, project proposal, timeline, work breakdown structure and project risk analysis. These along with context specific concerns such as closing physical spaces, communication, staffing and licencing issues impacted the successful completion of the project. The hospital libraries were closed and transitioned to a virtual library service within a six-month period. The new virtual library service launched in January 2018 offering document delivery, literature searching, online training and access to electronic resources licensed for health authority staff.

**Outcomes:**

Lessons learned during the transition to a virtual library service are shared to provide support for others considering, planning or actively undergoing a similar transition.

**Discussion:**

No librarian wants to close one library let alone several. Budgetary factors pressure health sciences libraries to adapt to new fiscal realities. In the health sciences, online availability and patrons desire for access at the bedside result in the need for libraries to respond to patron driven needs. A virtual library service is one response to the alignment of these factors.

## Introduction

The formation of the University of Manitoba Health Sciences Libraries (UMHSL) was the result of signing consecutive agreements over a period of twenty-four years between the University of Manitoba (UM) and Winnipeg area hospitals, now collectively known as the Winnipeg Regional Health Authority (WRHA). In 2017, the UMHSL included the Health Sciences Library (HSL) and eight hospital and health centre libraries located in the city of Winnipeg. Funding for library services to hospitals and health centres was provided to the UM as a transfer of baseline funds from the provincial government. Changes in the use of library services, technology and budgetary concerns were the prime influencers in the decision to close the hospital and health centre libraries. The use of hospital library spaces and services was trending downward in most respects. Increasing demand for remote electronic access by health care professionals outside of hospitals, and a flat budget supporting the agreements with the WRHA, required a substantial change in library service. In 2018, all the hospital and health centre libraries closed and the University of Manitoba Libraries (UML) opened the rebranded *WRHA Virtual Library*. This article describes the complications and lessons learned while closing the hospital libraries and opening a virtual library service to a distributed health care system with diverse clinical and educational needs.

The closure of hospital libraries is a noteworthy trend taking place across North America. According to Thibodeau and Funk, in 1989, 44% of United States (US) hospitals had on-site libraries, and by 2006 the percentage of hospitals with libraries had dropped to between 29.1% and 33.6% [1]. Recently, Harrow et al. examined the Library Book and Trade Almanac and determined that there was a 30% decrease in US medical libraries between 2007-2017 [2]. In their benchmarking study of Canadian health facility libraries by Ducas et al. [3] the authors identified 250 health facility libraries for study. Since the time of the Ducas et al. publication, Neame [4] and Hurrell et al. [5] wrote about the closure of hospital libraries in British Columbia and Alberta respectively. Thibodeau and Funk also provide data (October 2006 – May 2008) from the Medical Library Association's *Change in Status* form. They noted that twenty-two libraries closed, eight libraries merged, seven libraries merged with other hospital departments, and seven libraries became virtual libraries [1]. This data shows that while library closures are common, libraries are embracing other service models as an alternative to closing completely.

During the summer of 2017, the UML and the WRHA reached an agreement to close all eight hospital libraries in favour of a virtual library service model. The final result was the centralization of collections, services, and staff at the HSL, and the *WRHA Virtual Library* launched on January 21, 2018.

## Description

Thibodeau and Funk [1], identify many factors influencing the closure of physical libraries, including economics and changes in technology, both of which were critical factors in the closure of the hospital libraries in Winnipeg. Specifically, a flat budget could not accommodate increasing hospital library staff salaries and electronic resource licensing costs. Changes in the use of the physical library spaces and collections were also significant factors in transitioning the hospital libraries to the virtual library.

Library staff salaries predictably change with the cost of living and collective agreements for unionized staff. Typical hospital library staffing included at least one full-time faculty-status librarian, and at least one credentialed library assistant all with continuing appointments. At peak staffing levels, there were 10 librarians and 12.5 library assistants supporting the hospital libraries. Successful promotions in rank resulted in increased salary costs for librarian positions. Successful applications for research and study leaves required hiring additional staff to ensure the continuity of library services to the WRHA.

As the technology evolved during the agreements, so too did the definition of “access to collections.” In 1994, when the first agreements were signed electronic access to monographs and serials was very limited at the UML. Originally, “access to collections” was understood to mean access to physical collections. As electronic access to serials and monographs became ubiquitous around the middle 2000s agreements signed at this time required, a reinterpretation of “access to collections” to include all electronically available collections at the UML. This reinterpretation required license renegotiation to include WRHA patrons. Including this group of users in University-facilitated electronic licenses resulted in substantial costs to the UML over time. Adding pressure to the situation, WRHA clinical staff not covered under the agreements demanded the same access to electronic resources enjoyed by eligible WRHA patrons.

The use of the hospital library, physical spaces, collections and services declined over time. Circulation statistics from print collections between 2010 through 2017 showed a negative percent change every year over the previous year. Percent changes were from as little as - 4.1% to as much as -34.4% with the exception of a modest 1.1% increase in the final year. The trend clearly showed a reduction in the use of print collections. In addition, further analysis of circulation statistics from 2017 showed that less than 11% of the hospital libraries physical collections circulated. Literature search requests gradually declined over six years by 54% from the peak request year in 2011. Between 2010 and 2017, document delivery requests declined by 79% and reference questions by 54%.

### Project Management

This project was complex with many moving parts, necessitating periodic priority and timeline adjustments. The decommissioning process was completed within six months (November 2017 - May 2018) using standard project management processes. These processes included stakeholder consultation with library staff and WRHA administration, the organization of a project team, the development of a project proposal, charter and plan, timeline, work breakdown structure and project risk analysis.

UML administration consulted library staff regarding their recommendations for the future of the WRHA library service, resulting in a proposal for the Virtual Library. UML administration established a project team consisting of the author and an Associate University Librarian. The author drafted a project plan outlining the scope of the project, responsibilities, reporting structure, and work breakdown structure, which received approval from UML administration in November 2017. The plan included details concerning the removal of collections, furniture, equipment, and supporting the relocation of staff. The HSL staff established parameters for a rigorous deselection project and identified space to receive anticipated collections, furniture, equipment, and staff. At the same time, hospital library staff deselected collections, reviewed files for retention, and undertook inventories of furniture and equipment. The gradual, staged closure and turnover of libraries proceeded in priority order as established by the project plan.

### Communication

Communication with stakeholders during times of change is a crucial concern in project management. The project team carefully identifies the stakeholders and ensures that they are kept appropriately informed at all points during the project. Given that there were two organizations involved in this project, communication was both critical and extremely challenging.

The stakeholders identified included: library staff within the hospitals; library staff within the UML; information services and technology; administrative and facilities management staff at the University and within the hospitals; document delivery partner libraries; library vendors; moving companies; an offsite storage company; and last but certainly not least, the patrons. Each group required different forms and types of communication tailored to their needs at different times during the project.

Open communication with library staff contributed to staff engagement and buy-in with the project. Hospital library staff kept the project team apprised of any issues which might affect the project's timeline and provided frequent progress reports. Regular meetings, on-site visits and email and phone communication resulted in all library staff members involved in the project being on the same page at all times.

The project team was able to communicate freely with all stakeholders except for hospital library patrons. Either UML administration or the WRHA's administration provided all communication to hospital staff regarding the project. The announcement of the closure of the hospital libraries and the opening of the *WRHA Virtual Library* was jointly delivered by memo on October 16, 2017. The memo informed WRHA staff about the closure of the hospital libraries and the opening of the new virtual library, but did not include details regarding timelines or process, as this information was not available at the time of the announcement. Three months later, on January 29, 2018, the UML sent an email to all eligible WRHA staff announcing their access to the new virtual library service. In this system generated email eligible patrons were given their user ID and password for remote access to resources, provided links to the new virtual library web page and given contact information for the service. Five months after the original announcement, on April 2, 2018, the UML sent an email to all old region borrower accounts with a notice of the discontinuation of the accounts and reiterating that access is now only available through the virtual library. These were the only communications regarding the project provided to WRHA patrons between October 2017 and May 2018. This communication strategy was an attempt to control the message; managing the potential for misinformation and the possibility of negative sentiment from patrons. Even with this restriction in place, news of the closures appeared in the local newspaper [6]. Controlling the message while common practice in project management also requires frequent communication with stakeholders similar to that outlined by Neame [4].

In a further attempt to control the message, hospital library staff were not authorized to communicate with their patrons about the closures. This communication restriction created significant barriers to the completion of the project. This lack of communication left WRHA patrons confused about how to access library spaces, their collections, services, and how to return materials currently on loan from hospital library sites. Informing hospital library patrons of these practicalities was assigned to the virtual library staff and comprised most of the initial questions received after the opening in January 2018.

### Opening the Virtual Library

Long before the opening of the virtual library the UMHSL had all the necessary infrastructure in place to deliver library services electronically to patrons. Virtual library service offerings included document delivery, reference and research services, literature searching, and remote bibliographic instruction. The two areas which required attention before the opening were staffing and electronic licensing for WRHA patrons.

### Staff

From 2016, when negotiations opened between the UML and WRHA, contract staff occupied the majority of open and available positions within the university library system. Due to earlier planning and identification of possible future needs for the placement of displaced hospital library staff, there were no job losses. At the close of the hospital libraries, there were 6 librarians and 7.5 library technicians and assistants. UML administration identified 7 and 7.5 placements, respectively, within the system for librarians and support staff. These placements included four librarians and four support staff for the new virtual library.

Separate unions' collective agreements cover professional librarians and support staff at the University of Manitoba. The support staff collective agreement was prescriptive and provided clear guidance to libraries administration regarding relocation of staff based on seniority. The collective agreement pertaining to professional librarians is the same as for faculty at the UM. As such, it does not include language for the relocation of staff. Libraries administration, the University’s human resources department, and union representatives developed a process for librarians to select available positions.

Upon opening, the virtual library had one staff member physically located in the new offices and six others operating from still-open hospital libraries. This situation was not ideal but remained in place until all libraries were finally closed in March 2018.

### Licensing

All electronic resources licensed at the UML previously included access for a limited number of WRHA patrons, including all staff located in hospitals, and WRHA corporate offices. These licenses included those resources not typically utilized in a health care environment. In 2016-2017 the UML reported [7] expending $11,537,190 in Canadian funds (CAD) on ongoing resource purchases such as electronic licenses and subscriptions. Negotiating one license for two different organizations was a complicated and expensive endeavour as different methods to determine cost are used for each organization. For example, licences for hospitals are based on the number of beds while for academic institutions they are typically based on the number of simultaneous users required. Increasing licensing costs for the electronic resources desired by the WRHA resulted in costs pushing past the boundaries of the original budget for the reconceptualized WRHA library service.

Consultation with hospital librarians, the WRHA's administration, and vendors informed the selection of the initial offering of a much-reduced subset of electronic resources for the virtual library. On opening day, the WRHA Virtual Library reported [8] expending $738,000 (CAD) on electronic licenses and subscriptions and within budget. This suite of licensed resources changes in response to a variety of factors, including patron demands, budget, and cost per use analysis. Licensed resources now support a much broader group of eligible WRHA library patrons, including hospital staff, corporate office staff and WRHA primary care staff located outside the hospitals as identified by the WRHA (approx. 20,000), who may access electronic resources from where ever they live or work.

## Outcomes

While the intent of this paper is not to evaluate the virtual library service, summary statistics (Table 1) provide a glimpse of the changes resulting from this transition. Note that there was no formal program of promotion for the new service during the time frame of the statistics. While most service offerings remained the same and can be counted the same way some services were delivered differently. For example, training migrated from in-person training to online only. The numbers provided in the table include the total number of in-person sessions offered in 2016-2017 and the total number of views of webinars and training videos in 2018-2019. In addition, web services were offered from separate unit pages for each hospital in 2016-2017 and a single unified service page in 2018-2019.

**Table 1:** Summary statistics for select library services.

Service	Hospital Libraries 2016-2017	WRHA Virtual Library 2018-2019
Clients with cards	5000+	14,222
Reference Questions	5741	2324
Literature Searches	709	309
Systematic Reviews	9	4
Document Delivery	953	1855
Training	239	625
Website statistics	34,224	80,852

The effectiveness of project management processes to close the hospital libraries and open the *WRHA Virtual Library* are anecdotal. The project's closing document did solicit and receive feedback from library staff regarding the process. However, due to project time constraints, the author did not seek Research Ethics Board approval; thus, this information is not available for publication. Establishing a work break down structure, with milestones, dependencies, tasks and timelines enabled the author to consider all project changes within context. As risks to the project were identified, they were dealt with within the project plan. This allowed for timely adjustments to tasks and milestones ensuring the project was completed as efficiently as possible.

The closure of the hospital libraries was an extremely stressful experience for library staff. Open and frequent communication between the project team with library staff regarding goals, tasks, timelines and progress ensured everyone knew what was happening when and that their contribution was valued. Particularly effective were the use of site visits and face to face meetings allowing the library staff an open forum to voice concerns, identify problems and recommend possible solutions. It’s only through open communication with library staff that the project was completed in the timeframe given. Less successful in this project was communication with patrons. A more complete communication strategy could have alleviated patron concerns about the closure of the hospital libraries and the continuity of library services. In addition, a post-transition survey of clients may have been useful to highlight their perspective on the process but this was not developed. Anecdotes from virtual library staff indicated that clients were confused and sometimes frustrated about what access they had, what services they were eligible for and how to access what they did have.

## Discussion

The closure of the hospital libraries and the opening of the virtual library took place over six months. The application of project management processes and transparent communication with library staff are the primary reasons for the timely completion of the project.

The project plan required altering several times to address shifting priorities and deadlines. After the WRHA announced to hospital administrators that libraries were closing, some administrators came forward with plans to repurpose library space. In some cases, plans were time-dependent and necessitated a revision of the hospital library closing dates. Using the project plan meant the project team did not have to rethink all aspects of the project to address changes. The critical aspects of the project were archived in the project plan and considered in any adjustment.

Successful project management requires careful and clear communication [4], both formal and informal, with all stakeholders. While the closure of the hospital libraries was politically sensitive for both organizations involved, this needed balancing with the practical aspects of closing eight hospital libraries. Leveraging the long-standing relationships developed by hospital library staff with their patrons and using formal consistent and clear communication during the closures could have smoothed over the patrons' transition to the virtual library. The communication restriction created unnecessary barriers to the completion of project tasks and likely fostered negative sentiment from Region library patrons instead of mitigating it.

Staff relocation in a unionized environment requires significant time and consideration in a project of this nature. Early discussions with union representatives, human resources and other stakeholders are necessary to facilitate the process. Relocating staff during a project can affect project priorities and timelines, and this requires planning different response scenarios to put in place as needed.

Flat library budgets often cannot accommodate increasing hospital library staff salaries and electronic resource licensing costs. The virtual library at opening was staffed with four librarians and four library assistants a substantial reduction from peak staffing numbers. The UML reduced license costs for non-relevant materials by licensing relevant to context resources specifically for WRHA staff. Closure of physical spaces reduced the operating budget for this service. In this case, the Virtual Library was a cost-effective and technically viable alternative response to closing physical libraries and services completely.
